# Effect of Residual and Transformation Choice on Computational Aspects of Biomechanical Parameter Estimation of Soft Tissues

**DOI:** 10.3390/bioengineering6040100

**Published:** 2019-10-29

**Authors:** Ankush Aggarwal

**Affiliations:** Glasgow Computational Engineering Centre, School of Engineering, University of Glasgow, Glasgow G12 8LT, UK; ankush.aggarwal@glasgow.ac.uk

**Keywords:** biomechanics, parameter estimation, nonlinear preconditioning, gradient-based minimization, cirrus

## Abstract

Several nonlinear and anisotropic constitutive models have been proposed to describe the biomechanical properties of soft tissues, and reliably estimating the unknown parameters in these models using experimental data is an important step towards developing predictive capabilities. However, the effect of parameter estimation technique on the resulting biomechanical parameters remains under-analyzed. Standard off-the-shelf techniques can produce unreliable results where the parameters are not uniquely identified and can vary with the initial guess. In this study, a thorough analysis of parameter estimation techniques on the resulting properties for four multi-parameter invariant-based constitutive models is presented. It was found that linear transformations have no effect on parameter estimation for the presented cases, and nonlinear transforms are necessary for any improvement. A distinct focus is put on the issue of non-convergence, and we propose simple modifications that not only improve the speed of convergence but also avoid convergence to a wrong solution. The proposed modifications are straightforward to implement and can avoid severe problems in the biomechanical analysis. The results also show that including the fiber angle as an unknown in the parameter estimation makes it extremely challenging, where almost all of the formulations and models fail to converge to the true solution. Therefore, until this issue is resolved, a non-mechanical—such as optical—technique for determining the fiber angle is required in conjunction with the planar biaxial test for a robust biomechanical analysis.

## 1. Introduction

Characterizing the biomechanical properties of soft tissues remains a crucial starting point for describing and predicting their behavior [1]. Different experimental techniques have been designed, and several decades of research has produced a large amount of stress-strain data for different tissue types, such as aorta [2], myocardium [3], and heart valves [4,5]. Unlike engineered materials, most of these tissues exhibit highly nonlinear and anisotropic responses. In order to describe the wealth of experimental data, different constitutive models have been developed, and nonlinearity and anisotropy remain a hallmark of these models [6].

With the increasing complexity of the constitutive models for soft tissues, the number of associated fitting parameters has also increased. The process of fitting these models to the experimental data, also knowns as parameter estimation, is an important step [7]. It has been observed that reliably estimating the parameters can be a challenge, especially as the number of unknown parameters increase [8]. Moreover, due to the high nonlinearity and anisotropy, the parameters can become correlated, and the experimental data may not be sufficient to uniquely identify them [9,10]. In fact, determining an optimum set of experiments required to uniquely and accurately estimate the model parameters is an active area of research [11].

On the other hand, the effects of parameter estimation techniques on biomechanical characterization remain under-analyzed. Generally, methods originally designed for estimating parameters in linear and isotropic models are used as is, which can suffer from ill-conditioning and slow convergence when applied to the highly nonlinear problems of tissue mechanics. More importantly, in many cases the uniqueness of the estimated parameters cannot be tested, which may result in dubious outcomes. Similarly, extensive research has been carried out in improving the conditioning of linear algorithms [12]; however, these techniques for linear problems may not benefit the nonlinear parameter estimation common in biomechanics. Thus, there is a strong need for advancement in the area of nonlinear parameter estimation to help resolve these issues.

Previously, using simplified analysis and elementary algebra arguments, modifications were proposed to improve the biomechanical parameter estimation [13]. However, that study was focused solely on the case with two unknowns, which has only one local minima. In other words, for two parameters, the iterations always converged to the correct solution, and the improvement was obtained in the speed of convergence. Moreover, one of the proposed modifications was to use a logarithm of the measured stresses, which poses a problem if the stresses are negative. Thus, there is a need to further develop these techniques that are more general and easily applicable.

The aim of this study is to thoroughly test novel techniques in the parameter estimation process for multiple unknowns. To aid easy adaptation and wide applicability, the presented work is restricted to only simple modifications that are straightforward to implement and focus on the issue of non-convergence. In Section 2, details of the methods used are described: how artificial experimental data is generated, which constitutive models are used, details of the algorithm used for parameter estimation, and the transformations tested. In Section 3, results for each model and different formulations are presented. At the end, in Section 4, the significance of the results, limitations and possible future work are discussed before concluding in Section 5.

## 2. Methods

### 2.1. Problem Setup

In order to make this study relevant to experimental situations, planar biaxial test protocols—both displacement controlled (DC) and force controlled (FC)—are used. Strain/stress ratios of 0:1, 1:1, and 1:0 in the x– and y–directions are applied. Although, in practice, five or seven stress-strain ratios are used, as using more data is expected to improve the accuracy of the fitted model. That is true when there is noise present in the data and/or the model is not perfect. However, in this study the experimental data is assumed to be noiseless and perfect, which makes the parameter estimation easier and theoretically only two stress-strain ratios are required to estimate all the parameters uniquely. Lastly, shear stresses and deformations are neglected, and plane stress and incompressibility assumptions are used. Thus, only two stress-strain relations remain relevant.

In the DC case, the inputs are stretch ratios $\lambda_{x}$ and $\lambda_{y}$, and the outputs are Cauchy stresses $\sigma_{xx}$ and $\sigma_{yy}$. Inversely, in the FC case, the inputs are Cauchy stresses $\sigma_{xx}$ and $\sigma_{yy}$, and the outputs are stretch ratios $\lambda_{x}$ and $\lambda_{y}$. The maximum axial stretch applied is 1.1225, while the maximum normal stress applied is 130 kPa. The material parameters are assumed to be homogeneous everywhere, and hence for the DC case, the input-output relation is analytical. Moreover, even for the FC case, an iterative solver based on the Powell hybrid method [14] is used to calculate stretch ratios from the stresses. Thus, no finite element simulations are required, which helps keep the computational expenses reasonable.

### 2.2. Constitutive Models

Standard notations in large deformation mechanics are adopted [15]: F is the deformation gradient, $C=F^{⊤}F$ is the right Cauchy-Green deformation tensor, and $I_{1}=\mathrm{tr}\left(C\right)$ is the first invariant of C. Due to incompressibility, the Jacobian of the deformation J=detF is constrained to be unity J=1. If the fiber direction is denoted by a direction vector M, the stretch along the fiber is defined by the fourth invariant of C: $I_{4}=M\cdot CM$. For planar tissues with in-plane fibers aligned at an angle θ to the x-axis and normal direction parallel to the z-axis, the fiber direction can be written as $M={\left[\cos(\theta ),\sin(\theta ),0\right]}^{⊤}$.

The Cauchy stress tensor is σ, which is derived from the strain energy density Ψ as

(1) $$\sigma =2F\cdot \frac{\partial \Psi }{\partial C}\cdot F^{⊤}-pI,$$

where I is the identity tensor and *p* is the hydrostatic pressure acting as a Lagrange multiplier to enforce incompressibility. To define the stress-strain relationship, the following four constitutive models popular in the biomechanics community are used; however, different symbols are used for the material parameters than the literature for consistency across the models in this study.

#### 2.2.1. Gasser-Ogden-Holzapfel (GOH) Model

The GOH model defines the strain energy density function as [16]

(2) $$\Psi \left(C\right)=\frac{c_{1}}{2c_{2}}\left(e^{Q}-1\right)+\frac{c_{0}}{2}\left(I_{1}-3\right),$$

where

(3) $$Q=c_{2}{\left[c_{3}I_{1}+\left(1-3c_{3}\right)I_{4}-1\right]}^{2}.$$

The first term is the contribution of fibrous tissue, whereas the second term is assumed to be due to isotropic matrix. $c_{J}$ (J=0,1,2) are material parameters, and the dispersion parameter $c_{3}\in \left[0,1/3\right]$ and the fiber angle $c_{4}\equiv \theta \in \left[0,\pi \right]$ are the structural parameters. Thus, GOH model has a total of M=5 constitutive parameters.

#### 2.2.2. Humphrey Model

Humphrey and Yin proposed the following strain energy density function for soft tissues [17]

(4) $$\Psi \left(C\right)=\frac{c_{1}}{c_{2}}\left[e^{c_{2}\left(I_{1}-3\right)}-1\right]+\frac{c_{3}}{c_{4}}\left[e^{c_{4}{\left(\sqrt{I_{4}}-1\right)}^{2}}-1\right].$$

Here, $c_{J}$ (J=1,2,3,4) are material parameters, and the fiber angle $c_{5}\equiv \theta \in \left[0,\pi \right]$ is the structural parameter. Since there is no neo-Hookean term with parameter $c_{0}$, the Humphrey model also has M=5 constitutive parameters.

#### 2.2.3. Lee–Sacks Model

Lee et al. proposed the following constitutive model for valve tissue [9]:

(5) $$\Psi \left(C\right)=\frac{c_{0}}{2}\left(I_{1}-3\right)+\frac{c_{1}}{2}\left(c_{4}e^{c_{2}{\left(I_{1}-3\right)}^{2}}+\left(1-c_{4}\right)e^{c_{3}{\left(I_{4}-1\right)}^{2}}-1\right).$$

Here, $c_{J}$ (J=0,1,2,3) are the material parameters, and the fiber angle $c_{5}\equiv \theta \in \left[0,\pi \right]$ and $c_{4}\in \left[0,1\right]$ are the structural parameters. Thus, Lee–Sacks model has M=6 constitutive parameters.

#### 2.2.4. May-Newman Model

May-Newman and Yin proposed the following strain energy density to define the biomechanical properties of mitral valve tissue [18]

(6) $$\Psi \left(C\right)=c_{1}\left(e^{Q}-1\right)+\frac{c_{0}}{2}\left(I_{1}-3\right),$$

where

(7) $$Q=c_{2}{\left(I_{1}-3\right)}^{2}+c_{3}{\left(\sqrt{I_{4}}-1\right)}^{4}.$$

Here, $c_{J}$ (J=0,1,2,3) are material parameters, and the fiber angle $c_{4}\equiv \theta \in \left[0,\pi \right]$ is the structural parameter. Thus, May-Newman model has a total of M=5 constitutive parameters.

### 2.3. Parameter Estimation Algorithm

A general parameter estimation problem for planar biaxial tissues is the following: given the “measured” values of stresses and stretches and a chosen constitutive model, determine the associated constitutive parameters $c=\left\{c_{0},c_{1},\cdots ,c_{M}\right\}$. The experimental input is denoted as $x_{i}$ and output as ${\bar{y}}_{i}$, i=1,⋯,n where *n* is the number of experimental data points. In the DC case, input $x_{i}=\left[\lambda_{xx}^{i},\lambda_{yy}^{i}\right]$ are the stretches and output ${\bar{y}}_{i}=\left[\sigma_{xx}^{i},\sigma_{yy}^{i}\right]$ are the stresses, whereas in the FC case, input $x_{i}=\left[\sigma_{xx}^{i},\sigma_{yy}^{i}\right]$ are the stresses and output ${\bar{y}}_{i}=\left[\lambda_{xx}^{i},\lambda_{yy}^{i}\right]$ are the stretches. The deviation of the chosen model from the measured output is defined as the residual

(8) $$r_{i}\left(c\right)=\langle m\left(x_{i};c\right),{\bar{y}}_{i}\rangle ,$$

where *m* is the input–output function derived using the chosen constitutive model and then evaluated at $x_{i}$ input and chosen c parameter values. The residual operator 〈·,·〉 needs to be chosen appropriately. A commonly used option is the uniformly weighted difference

(9) $$r_{i}^{U}=m\left(x_{i};c\right)-{\bar{y}}_{i}.$$

In [13], a “log-norm” was proposed which decreased the nonlinearity and improved the convergence. However, logarithm has a drawback that it cannot be applied to negative values. It should be noted that taking a log has the effect of assigning lower weights to higher values. In other words, $\log\left(y_{1}\right)-\log\left(y_{2}\right)=\log\left(y_{1}/y_{2}\right)$. Inspired by this observation, an alternative residual is tested: a non-uniformly weighted difference

(10) $$r_{i}^{N}=\frac{m(x_{i};c)}{{\bar{y}}_{i}}-1.$$

While using Equation (10), one must exclude points with measured zero values. In practice, this is easily implemented since exactly zero output is uncommon for anisotropic tissues (except in the load-free reference configuration, which is trivially satisfied by all models and can be simply discarded). Lastly, an objective function is defined (also called the Loss function in literature), as simply the square summation of the residual:

(11) $$F\left(c\right):=\frac{1}{2}\sum_{i=1}^{n}\left(r_{i}\cdot r_{i}\right).$$

In order to determine the parameters, a minimization problem is formulated to estimate the parameters $c_{J}$:

(12) $$\bar{c}=\underset{c}{arg min}F\left(c\right).$$

The minimization problem is solved using the Gauss-Newton algorithm with a backtracking line search [19]. Details are provided in Algorithm 1.

Since the parameter space size grows exponentially with the number of parameters, it becomes prohibitively expensive to systematically span the space for more than three parameters. Therefore, Latin Hypercube Sampling (LHS) is used to generate 300 samples from the parameter space (see Table 1 for the parameter ranges used). LHS has the advantage of generating uniformly spaced combinations of the parameters while keeping the parameter values random. Using each LHS sample $C^{k}$ (k=1,⋯,300), artificial “measurements” are generated as ${\bar{y}}_{i}=m\left(x_{i},C^{k}\right)$. Thus, the “true” parameter values are known, and the estimated results can be compared against them. Since nonlinear parameter estimation depends strongly on the initial guess, all other samples $C^{l}$ ∀l≠k are used as the initial guesses $c_{0}$ for the parameter estimation algorithm. Thus, for each test, 300×299 = 89,700 minimization simulations are computed. Finally, the histogram of the fraction of cases versus iterations taken to converge are plotted. To test the convergence, the final parameter values are compared with the true parameter values, and the error is calculated. Since the fiber angle θ is cyclic with a period of π, the value of $\cos^{2}\left(\theta \right)$ is compared instead. The non-converged cases are subcategorized into “Unconverged” (U) and “Misconverged” (M): U being the runs that were unable to converge in maximum number of iterations and M being the ones where minimization converged, however, to wrong parameter values.

**Algorithm 1:** Parameter estimation using Gauss-Newton method with backtracking line search. **Data**: Observed data $\bar{y}$ and initial guess $c_{0}$, MAXITER=30, $TOL=10^{-10}$, $\delta =10^{-5}$
 **Result**: Parameters that fit the model y to observed data $\bar{y}$ by minimizing the functional
    $F\left(c\right)=\frac{1}{2}r\cdot r$ (11) with the chosen residual (9) or (10)
 initialization $c\leftarrow c_{0}$;
 ITER←0;
 
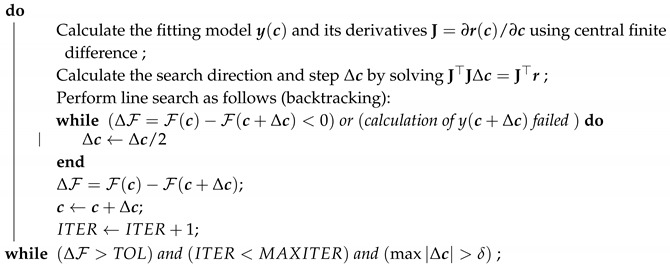


### 2.4. Parameter Transformations

The aim is to study the effect of transforming parameter space from c to $\hat{c}=\Gamma \left(c\right)$ on the minimization. As the first step, linear parameter transformation is tested, and, in general, a linear transformation can be written as $\hat{c}=Pc$, where P is a constant matrix. A natural simple linear transformation is rescaling the parameters

(13) $$\hat{c}=\left[\eta_{1}c_{1},\cdots ,\eta_{M}c_{M}\right],$$

so that the derivative of the residual $\partial F/\partial {\hat{c}}_{J}$ are of the same order. That is,

(14) $$\eta_{J}=\sum_{i=1}^{n}r_{i}\frac{\partial r_{i}}{\partial c_{J}}$$

calculated from the previous iteration. It should be noted that this rescaling transformation is equivalent to the well-established Jacobi preconditioner [12].

Next, nonlinear transformations are tested. As shown in [13], taking a log of the parameter $c_{1}$, i.e., ${\hat{c}}_{1}=\log\left(c_{1}\right)$ was shown to accelerate the convergence for estimating two unknown parameters. To test if this holds true for different models and multiple parameters, the following transformation is used

(15) $$\left[{\hat{c}}_{1},{\hat{c}}_{2},{\hat{c}}_{3},\cdots \right]=\left[\log\left(c_{1}\right),c_{2},c_{3},\cdots \right].$$

Since the model by Humphrey (4) has sum of two exponential terms, an equivalent transformation is tested

(16) $$\left[{\hat{c}}_{1},{\hat{c}}_{2},{\hat{c}}_{3},{\hat{c}}_{4}\right]=\left[\log\left(c_{1}\right),c_{2},\log\left(c_{3}\right),c_{4}\right].$$

Using each transformation, the number of unknown parameters are gradually increased and their effect on convergence is tested. Lastly, Zhang et al. [20] proposed the following transformation:

(17) $$\left[{\hat{c}}_{1},{\hat{c}}_{2},{\hat{c}}_{3},\cdots \right]=\left[c_{1}e^{Q_{\max}\left(c\right)},c_{2},c_{3},\cdots \right],$$

where $Q_{\max}\left(c\right)$ is maximum value of the exponent over all applied inputs. Although this is not a simple transformation to implement, for comparison purposes, models with a single exponential term are tested for the DC case.

## 3. Results

Using linear transformation, there is no effect on the minimization process for any cases (results skipped for brevity). This is not surprising since the number of unknowns in this case is always less than six, which means that the Hessian matrix—even if it has a high condition number—can be inverted with high precision. Thus, the linear transformation has no effect on the presented problem, but it may improve the estimation for heterogeneous problem or while using an algorithm like the steepest-descent where the Hessian is not inverted. Henceforth, only the nonlinear transformations are focused on for different models described in the previous section.

### 3.1. GOH Model

**Figure 1:**
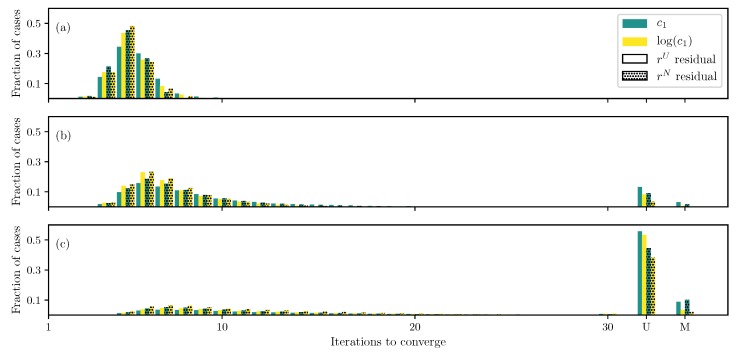
Iterations required using different formulations for GOH model (2) displacement controlled (DC) case when (**a**) only $c_{1}$ and $c_{2}$ are unknown, (**b**) all parameters except the angle θ are unknown, and (**c**) all parameters are unknown.

For the DC case with GOH model (2), as the first check, only two parameters, $c_{1}$ and $c_{2}$, are estimated while keeping other parameters fixed. Both the log transformation (15) and non-uniformly weighted residual (10) show faster convergence compared to the standard uniform weighted residual and no transformation (Figure 1a). Furthermore, the algorithm is able to find true parameters for all runs and formulations.

If the number of unknowns is increased to include $c_{0}$ and $c_{3}$ (but keep the fiber angle θ fixed), using the standard formulation (uniform weighted residual and no transformation), there are a small fraction of cases that either do not converge or converge to the wrong solution (Figure 1b). These cases are reduced to almost none when using the log transformation (15) and non-uniformly weighted residual (10). This is an important improvement in addition to faster convergence.

Furthermore, if the fiber angle θ is also treated as an unknown, the number of unconverged and misconverged runs increases dramatically, and only less than a third of the runs converge to the true parameter values (Figure 1c). Although, the log transformation (15) and non-uniformly weighted residual (10) improve the situation slightly, there still remains a large number of non-converged runs.

**Figure 2:**
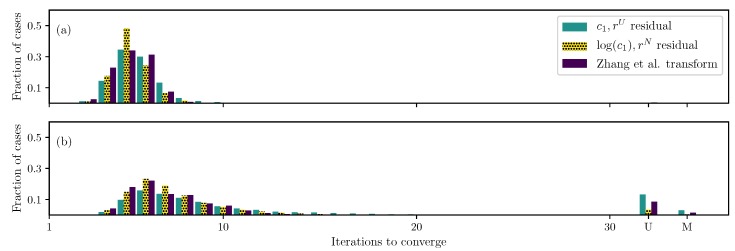
Iterations required using different formulations for the GOH (2) model DC case when (**a**) only $c_{1}$ and $c_{2}$ are unknown and (**b**) all parameters except the angle θ are unknown.

The standard formulation (uniformly weighted residual and original parameters) and the best formulation so far (non-uniformly weighted residual and $\log(c_{1})$ transformation) are compared with the one proposed by Zhang et al. [20] (Figure 2). Results show that Zhang’s transformation helps improve the convergence compared to the standard formulation; however, its performance is sub-par to the one proposed here—both for two and four unknown parameters.

**Figure 3:**
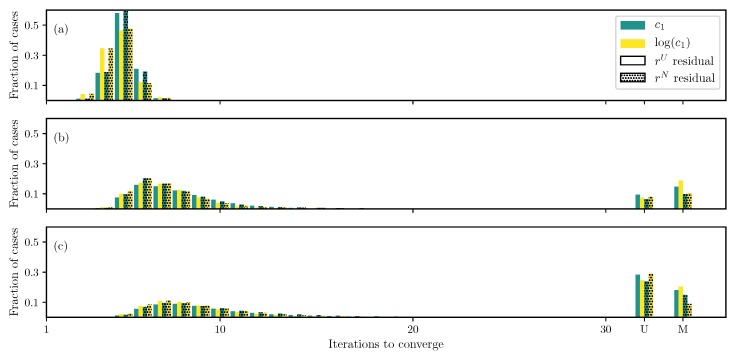
Iterations required using different formulations for the GOH (2) model force controlled (FC) case when (**a**) only $c_{1}$ and $c_{2}$ are unknown, (**b**) all parameters except the angle θ are unknown, and (**c**) all parameters are unknown.

For the FC case with the GOH model (2), as a start, only two parameters, $c_{1}$ and $c_{2}$, are estimated and all other parameters are fixed. Results show that all the cases are converged, and that using a log transformation improves the convergence speed (Figure 3a). However, compared to the DC case, no appreciable difference is found by using the non-uniformly weighted residual (10) in this case.

As the number of unknowns is increased to include $c_{0}$ and $c_{3}$, the behavior remains similar (Figure 3b). The log transformation helps the algorithm by making the convergence faster and decreasing the number of non-converged runs. However, there is no effect of the non-uniformly weighted residuals. If the fiber angle θ is also an unknown, similar to the DC case, the parameter estimation becomes extremely difficult, and only a small fraction of the runs converge (Figure 3c). There is only a small improvement by using the log transformation and non-uniformly weighted residuals. It should be noted that using Zhang’s formulation (17) for the FC case is problematic because the strain is unknown and finding the maximum value of the exponent will be expensive. Since no improvement was found in the DC case, the formulation by Zhang is omitted for the FC case.

**Figure 4:**
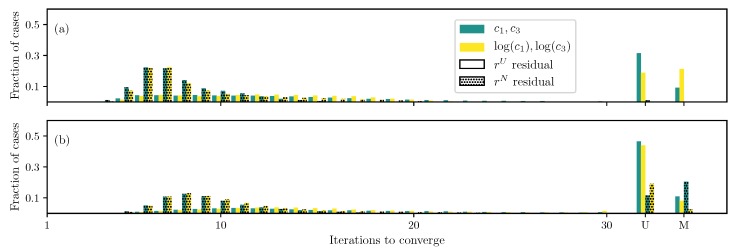
Iterations required using different formulations for the Humphrey model (4) DC case when (**a**) all parameters except the fiber angle θ are unknown and (**b**) all parameters are unknown.

For the DC case with standard formulation (uniform weighted residual and no transformation) and Humphrey’s model (4) with fiber angle θ fixed, there is a significant fraction of runs that are either unconverged or misconverged (Figure 4a). This is caused by the interaction of two exponential terms in the model. By using the log-log transform (16), the situation improves slightly. Furthermore, if the non-uniformly weighted residual (10) is also used, all of the cases converge. Thus, there is an enormous difference in convergence properties by using the transform and non-uniform weighting.

### 3.2. Humphrey Model

If the fiber angle also needs to be determined, the number of converged runs reduces substantially (Figure 4b). The misconverged simulations are reduced to almost none when the log transform is used; however, the number of unconverged cases increase. Thus, overall the total number of non-converged runs remains approximately the same, with only a slight improvement in the convergence using the log transformation (16). Since Humphrey’s model has two exponential terms, there is no simple method to find the maximum exponent for each term, and thus the formulation by Zhang et al. (17) is omitted.

**Figure 5:**
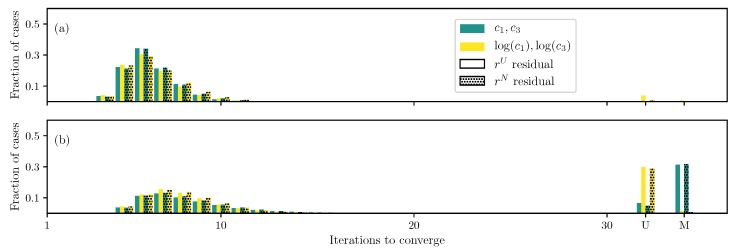
Iterations required using different formulations for the Humphrey model (4) FC case when (**a**) all parameters except the fiber angle θ are unknown and (**b**) all parameters are unknown.

A similar behavior for the FC case is found; when the fiber angle θ is fixed, the parameter estimation is successful for all runs. However, there is no appreciable improvement by using either the log transform or the non-uniformly weighted residual (Figure 5a). If the fiber angle θ is included as an unknown, it becomes challenging to estimate the parameters irrespective of the method used (Figure 5b). Although using non-uniformly weighted residual leads to a decrease in the number of misconverged runs, it also leads to an increase in the unconverged runs with only a slight increase in the number of converged runs.

### 3.3. Lee–Sacks Model

**Figure 6:**
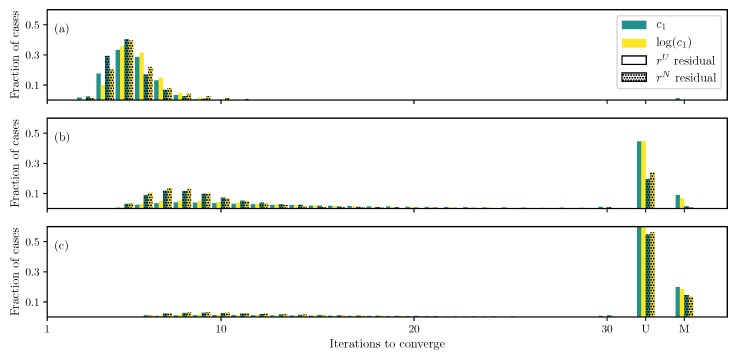
Iterations required using different formulations for the Lee–Sacks model DC case when (**a**) only $c_{1}$ and $c_{2}$ are unknown, (**b**) all parameters except the fiber angle θ are unknown, and (**c**) all parameters are unknown.

In Lee–Sacks model (5), when only two parameters, $c_{1}$ and $c_{2}$, are estimated with the DC case, all of the runs converge irrespective of the formulation (Figure 6a). Using log transformation on $c_{1}$ helps speed up the convergence, while using the non-uniformly weighted residual (10) has a limited effect.

If all parameters except the fiber angle θ are estimated, the uniformly weighted residual leads to a large number of unconverged and misconverged runs (Figure 6b). However, changing the residual to non-uniformly weighted one (10) helps improve the situation. When the fiber angle is also estimated, similar to previous two models, a large number of unconverged and misconverged runs are obtained (Figure 6c). However, unlike the previous models, there is only a limited improvement when the residual is changed or the log transformation is used. Similar to the Humphrey’s model, Less–Sacks model also has two exponential terms, however, with only one parameter $c_{1}$ in front. Thus, it is not clear how to implement the transformation proposed by Zhang et al. (17).

**Figure 7:**
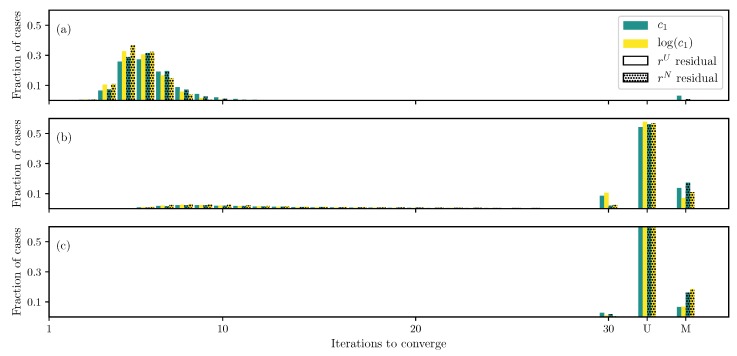
Iterations required using different formulations for the Lee–Sacks model FC case when (**a**) only $c_{1}$ and $c_{2}$ are unknown, (**b**) all parameters except the fiber angle θ are unknown, and (**c**) all parameters are unknown.

In the FC case with Lee–Sacks model, only two parameters, $c_{1}$ and $c_{2}$, can be reliably estimated using the standard formulation (Figure 7a). Using the log transformation and non-uniformly weighted residual speeds up the convergence. Furthermore, there is a small number of misconverged runs with only two unknowns using the standard formulation, which disappear when the log transformation is used. However, when the unknown parameters include other parameters, almost none of the FC runs are converged (Figure 7b,c). This happens irrespective of fiber angle θ being fixed or unknown and the choice formulation.

### 3.4. May-Newman Model

**Figure 8:**
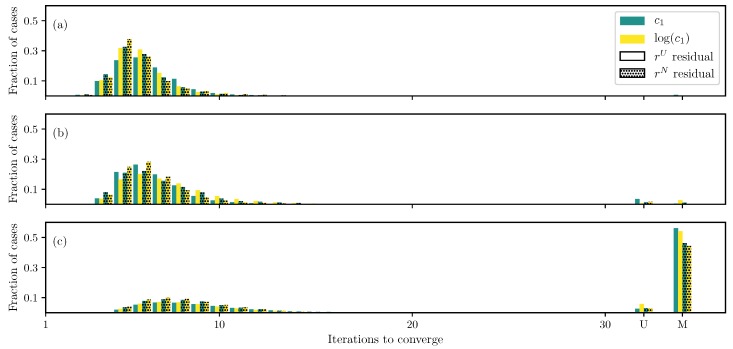
Iterations required using different formulations for the May-Newman model DC case when (**a**) only $c_{1}$ and $c_{2}$ are unknown, (**b**) all parameters except the fiber angle θ are unknown, and (**c**) all parameters are unknown.

Two parameters $c_{1}$ and $c_{2}$ in the May-Newman model (6) can be estimated using the DC setup and any of the formulations (Figure 8a). Using the log transformation and non-uniformly weighted residual helps improve the convergence speed. If the number of unknown parameters is expanded to include others, except the fiber angle, most of the runs converge to the correct solution (Figure 8b). The number of non-converged runs becomes lower if the log transformation and/or the non-uniformly weighted residual are used. However, the convergence speed is largely unaffected by the change in formulation.

When the fiber angle θ is included as an unknown, the same issue as other models appears where less than a third of the simulations converge to the correct solution (Figure 8c). By using the non-uniformly weighted residual, this problem is mitigated to some extent, although not completely. Furthermore, both the log transformation and non-uniformly weighted residual help reduce the iterations required to converge.

**Figure 9:**
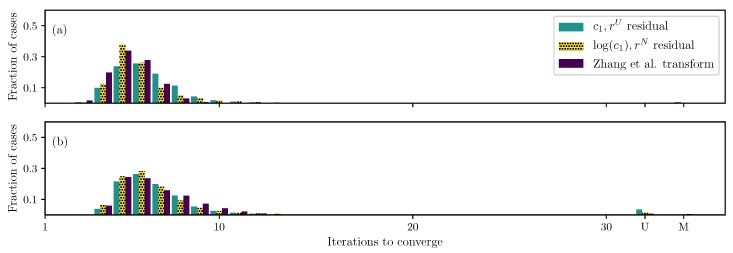
Iterations required using different formulations for the May-Newman (6) model DC case when (**a**) only $c_{1}$ and $c_{2}$ are unknown and (**b**) all parameters except the angle θ are unknown.

The results using Zhang’s transformation [20] are compared with the proposed formulations (Figure 9). Similar to the results for the GOH model, this transformation helps improve the convergence compared to the standard formulation. However, the improvement is less than that using the formulation proposed here.

**Figure 10:**
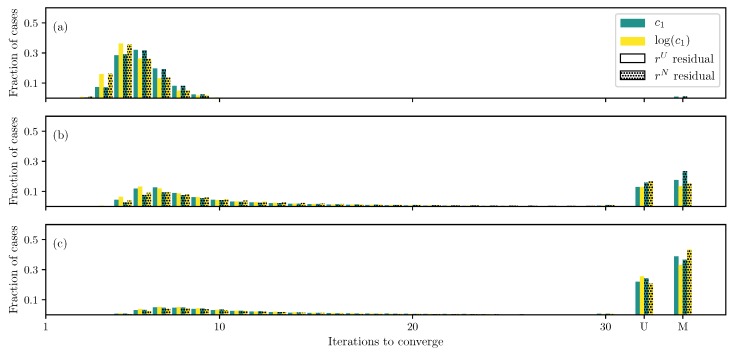
Iterations required using different formulations for the May-Newman model FC case when (**a**) only $c_{1}$ and $c_{2}$ are unknown, (**b**) all parameters except the fiber angle θ are unknown, and (**c**) all parameters are unknown.

Using the FC setup with May-Newman model, the convergence behavior is similar to other models. When only two parameters $c_{1}$ and $c_{2}$ are estimated, all runs converge (Figure 10a). In this case, the convergence speed is improved when the log transformation is used, but is unaffected by the residual choice. When all the parameters except the fiber angle θ are estimated, some simulations do not converge (Figure 10b). The number of misconverged runs is reduced slightly by using the log transformation, whereas using the non-uniformly weighted residual has a slight negative effect on the convergence. Lastly, when the fiber angle θ is also an unknowns, all the formulations suffer from poor convergence (Figure 10c). Only a small fraction of runs converge, and the choice of formulation has a negligible effect.

## 4. Discussion

### 4.1. Nonlinear Preconditioning

Linear transformation was found to have no effect on the convergence behavior. This is because the number of unknowns is small, and the tissue is assumed to be homogeneous. Thus, the Hessian matrix can be inverted accurately, and therefore linear preconditioners have no advantage for the presented problem. This is an important characteristic of biomechanical problems: the challenges are different from other engineering fields, which necessitates different solutions. The nonlinear transformation proposed here acts as a nonlinear preconditioner, which is a relatively under-explored area [21]. The improvements found in this study will motivate further work along these lines to improve the biomechanical parameter estimation and, therefore, analysis.

### 4.2. Replacing $c_{1}$ with $e^{{\hat{c}}_{1}}$

Across almost all models and cases, using a log transform of $c_{1}$ led to improvement in the parameter estimation. For many cases, it not only improved the convergence speed, but also helped decrease the fraction on non-converged simulations. It should be noted that taking a log of $c_{1}$ while parameter estimation is equivalent to replacing $c_{1}$ with $e^{{\hat{c}}_{1}}$ in the constitutive models. As noted in [13], this not only helps reduce the nonlinearity but also enforces the constraint $c_{1}>0$. Interestingly, not only this transform helped improve the DC cases, but it also helped improve the FC cases, albeit to a lesser extent. The improvements obtained are all the more impressive considering the minute nature of this change and its implementational simplicity.

### 4.3. Weighted Residual

Interestingly, the effect of non-uniformly weighted residual was similar to that of the “log”-norm proposed in [13]. It helped improve the parameter estimation for almost all DC cases, but did not have an appreciable—positive or negative—effect on the FC cases. The advantage of this approach over “log”-norm is that it can be used for both positive and negative values. Moreover, the non-uniformly weighted residual is already used in some optimization problems. However, this is the first time it is being compared with the uniformly weighted residual for biomechanical parameter estimation. The approach can be implemented easily and the results show a clear advantage over uniformly weighted residual.

### 4.4. Adding Fiber Angle as an Unknown

**Figure 11:**
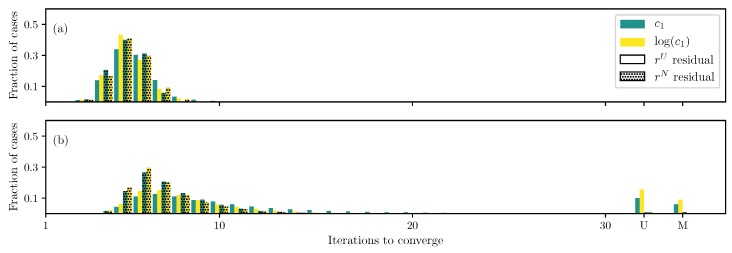
Iterations required using different formulations for (**a**) the GOH model DC case when all parameters except fiber angle θ and dispersion $c_{3}$ are unknown, (**b**) the Lee–Sacks model when all parameters except the fiber angle θ and dispersion $c_{4}$ are unknown.

It was not surprising that increasing the number of unknown parameters made their estimation more challenging for all models and cases. However, the most striking differences were observed when fiber angle was added to the unknown list; the fraction of converged results reduced drastically compared to when the fiber angle was considered as a known fixed value. More importantly, the use of log transform or non-uniformly weighted residual had an extremely limited effect when fiber angle was being estimated. Until this issue is resolved, the most practical approach may be to determine the fiber angle using other techniques, such as histology [22] or light scattering [23], and consider it as a fixed unknown during biomechanical parameter estimation. Using optical techniques, it may be possible to estimate the fiber dispersion, as well ($c_{3}$ in GOH model and $c_{4}$ in Lee–Sacks model). Not surprisingly, this will make the parameter estimation easier (Figure 11); however, the advantages of the log transform and non-uniformly weighted residual remain.

Furthermore, the ultimate goal of this study is to have a robust technique that can estimate all the parameters from in-vivo dataset, where inverse models are required [24,25,26]. Previously, in inverse model developed for the aortic valve, fiber angles had a significant effect on the solution accuracy [10], and therefore needed to be determined by other means. It should be noted that it may not be possible to determine fiber angle separately for in-vivo inverse models. In such situations, determining the population-averaged fiber architectures of native tissues would help make this possible [27,28]. In general, if the parameters can be split into separable subsets, where one subset of the parameters can be estimated before fitting the rest of them and the fitting the second subset does not affect the first one, the estimation process generally becomes easier and faster. However, this requires the two subsets must be theoretically separable. It should be noted that this is not the case for the tow region for any of the models used in this study, as the stiffness of the exponential part is non-zero even at zero deformation (F=I).

### 4.5. Displacement Controlled versus Force Controlled

The biaxial testing experimental protocols can be designed to be either displacement controlled or force controlled. In literature, there is some discussion on the implementation difficulty of these two approaches; however, there has been no study comparing them in terms of parameter estimation. The results presented here demonstrate that the two approaches have different convergence properties. For most of the problems, DC cases show better convergence than the FC cases. However, that is not always true, especially when the fiber angles are considered as unknowns. Furthermore, estimating parameters via FC setup requires solving the inverse of stress-strain relationship using an iterative solver, which adds to the computational expense. Therefore, if there is a choice, a DC setup may be easier from the parameter estimation point of view. However, to reliably estimate the fiber angle, FC setup might prove superior.

### 4.6. Limitations and Future Work

In this study, the focus was limited to simple modifications that are easy to implement. Thus, only a limited number of nonlinear transforms were tested, and those with a clear effect were presented. Although one could develop other nonlinear transforms, there is no clear method to find good candidates. This is especially important for determining the fiber angle, as an improvement is clearly needed in that area to make the parameter estimation robust. Similarly, this analysis could be done for newer models, such as one by Li et al. [29]. However, this model requires integration over a unit sphere for every stress calculation, which makes it computationally unfeasible to solve approximately 90 thousand minimizations. On the other hand, applying Latin Hypercube Sampling approach to Fung’s model [30] generates parameter sets with non-convex stress-strain relationship [31], and convexity is difficult to impose in the sampling process.

Due to the high computational cost of this study, the measured data $\bar{y}$ was assumed to be error-free (either modeling or noise). This is a limitation since the presence of error is likely to increase the chances of getting trapped in a local minima and increase the number of non-converged cases. Lastly, it may be possible to apply data-mining techniques on the non-converged cases and obtain insight into the non-convex nature of these parameter estimation problems. However, that is outside the scope of this manuscript, and these directions will be pursued in the future.

## 5. Conclusions

The aim of this study was to thoroughly analyze the effect of parameter estimation technique on biomechanical characterization of soft tissues using planar biaxial testing. Four invariant-based constitutive for soft tissues were tested, each with their own set of five or six parameters. Because of the large dimension of parameter space, Latin Hypercube Sampling approach was used to randomly generate parameter sets. These parameters were used as “target” parameter values, as well as initial guesses. It was found that small modifications of weighting the residual by experimental data and/or taking a log of the parameter in front of the exponential can significantly improve the parameter estimation process. The advantage was found not only in terms of convergence speed but also that the proposed modifications reduce the possibility of estimating wrong parameter values by getting stuck in a local minima. However, both the standard and modified formulations performed badly when fiber angle was considered as an unknown. Hence, the results suggest that determining the fiber angle using a non-mechanical test, as in, for example, an optical technique, can greatly help the parameter estimation process. Although, this may not be practical for in-vivo situations, for which further research is required to devise reliable parameter estimation techniques.

## Figures and Tables

**Table 1:** Summary of the models used and the ranges of associated parameters. GOH = Gasser-Ogden-Holzapfel model.

Model	$c_{0}$	$c_{1}$	$c_{2}$	$c_{3}$	$c_{4}$	$c_{5}$
GOH Equation (2)	0 to 100 kPa	5 to 100 kPa	20 to 100	0 to 0.3	0 to π	−
Humphrey Equation (4)	−	5 to 100 kPa	1 to 100	5 to 100 kPa	1 to 100	0 to π
Lee–Sacks Equation (5)	0 to 100 kPa	5 to 100 kPa	1 to 100	1 to 100	0 to 1	0 to π
May-Newman Equation (6)	0 to 100 kPa	5 to 100 kPa	1 to 100	1 to 1000	0 to π	−
